# Spleen Tyrosine Kinase Is a Critical Regulator of Neutrophil Responses to *Candida* Species

**DOI:** 10.1128/mBio.02043-19

**Published:** 2020-05-12

**Authors:** Paige E. Negoro, Shuying Xu, Zeina Dagher, Alex Hopke, Jennifer L. Reedy, Michael B. Feldman, Nida S. Khan, Adam L. Viens, Natalie J. Alexander, Natalie J. Atallah, Allison K. Scherer, Richard A. Dutko, Jane Jeffery, John F. Kernien, J. Scott Fites, Jeniel E. Nett, Bruce S. Klein, Jatin M. Vyas, Daniel Irimia, David B. Sykes, Michael K. Mansour

**Affiliations:** a Division of Infectious Diseases, Massachusetts General Hospital, Boston, Massachusetts, USA; b Center for Regenerative Medicine, Massachusetts General Hospital, Boston, Massachusetts, USA; c Department of Surgery, Massachusetts General Hospital, Boston, Massachusetts, USA; d Department of Medical Microbiology and Immunology, University of Wisconsin School of Medicine and Public Health, Madison, Wisconsin, USA; e Department of Pediatrics, University of Wisconsin School of Medicine and Public Health, Madison, Wisconsin, USA; f Division of Pulmonary and Critical Care Medicine, Massachusetts General Hospital, Boston Massachusetts, USA; g Department of Medicine, University of Wisconsin—Madison, Madison Wisconsin, USA; h Harvard Medical School, Boston, Massachusetts, USA; University of Zürich; Leibniz Institute for Natural Product Research and Infection Biology, Hans Knoell Institute Jena (HKI)

**Keywords:** neutrophils, spleen tyrosine kinase, *Candida*, fungus

## Abstract

Invasive fungal infections constitute a lethal threat, with patient mortality as high as 90%. The incidence of invasive fungal infections is increasing, especially in the setting of patients receiving immunomodulatory agents, chemotherapy, or immunosuppressive medications following solid-organ or bone marrow transplantation. In addition, inhibitors of spleen tyrosine kinase (Syk) have been recently developed for the treatment of patients with refractory autoimmune and hematologic indications. Neutrophils are the initial innate cellular responders to many types of pathogens, including invasive fungi. A central process governing neutrophil recognition of fungi is through lectin binding receptors, many of which rely on Syk for cellular activation. We previously demonstrated that Syk activation is essential for cellular activation, phagosomal maturation, and elimination of phagocytosed fungal pathogens in macrophages. Here, we used combined genetic and chemical inhibitor approaches to evaluate the importance of Syk in the response of neutrophils to *Candida* species. We took advantage of a Cas9-expressing neutrophil progenitor cell line to generate isogenic wild-type and Syk-deficient neutrophils. Syk-deficient neutrophils are unable to control the human pathogens Candida albicans, Candida glabrata, and Candida auris. Neutrophil responses to *Candida* species, including the production of reactive oxygen species and of cytokines such as tumor necrosis factor alpha (TNF-α), the formation of neutrophil extracellular traps (NETs), phagocytosis, and neutrophil swarming, appear to be critically dependent on Syk. These results demonstrate an essential role for Syk in neutrophil responses to *Candida* species and raise concern for increased fungal infections with the development of Syk-modulating therapeutics.

## Abstract (precis)

Neutrophils are recognized to represent significant immune cell mediators for the clearance and elimination of the human-pathogenic fungal pathogen *Candida*. The sensing of fungi by innate cells is performed, in part, through lectin receptor recognition of cell wall components and downstream cellular activation by signaling components, including spleen tyrosine kinase (Syk). While the essential role of Syk in macrophages and dendritic cells is clear, there remains uncertainty with respect to its contribution in neutrophils. In this study, we demonstrated that Syk is critical for multiple cellular functions in neutrophils responding to major human-pathogenic *Candida* species. These data not only demonstrate the vital nature of Syk with respect to the control of fungi by neutrophils but also warn of the potential infectious complications arising from the recent clinical development of novel Syk inhibitors for hematologic and autoimmune disorders.

## INTRODUCTION

Invasive fungal infections have become increasingly prevalent with associated high morbidity and mortality rates (1–3). Fungal pathogens typically occur in immunocompromised patients, particularly patients with solid-organ transplants or stem cell (SC) transplants or those receiving immunosuppressive therapies (4–6). The use of immunosuppressive therapy and invasive surgical procedures has also expanded among our patients who are older and who present with more-complex treatment requirements, increasing the scope of the population susceptible to infection (7, 8). *Candida* species can be found as commensals on the skin, vagina, and in the human digestive tract, yet remain harmless in most healthy hosts (9). However, in the setting of a compromised immune system, these same *Candida* species can become opportunistic pathogens and acquire invasive features resulting in morbidity and mortality (10, 11). Among the invasive infections caused by human-pathogenic yeasts, the majority are caused by *Candida* species, among which Candida albicans and Candida glabrata together account for approximately 90% of invasive cases in North America (4, 9, 11). More recently, Candida auris has emerged as a new species demonstrating high rates of drug resistance associated with significant mortality (12). First identified in Japan in 2009, C. auris has now emerged as a cause of severe illness and outbreaks in hospitalized patients around the world, including the United States (13, 14). High rates of drug resistance have presented clinical challenges and in turn resulted in significant mortality (15). In addition, C. auris persists on the surfaces in rooms and equipment of health care facilities despite standard cleaning procedures, increasing the risk of patient to patient transmission (14, 16).

Spleen tyrosine kinase (Syk) is a critical kinase that was first thought to be restricted to adaptive immunity but has since been shown to play crucial roles in innate immunity, including fungal immunity (17). Many cell surface receptors, such as Dectin-1, Dectin-2, complement, and Fc receptors, that participate in recognition of fungal components rely on Syk for downstream signaling (15, 18, 19). The central role of Syk in adaptive immune cell activation has made this kinase a promising target for the development of anti-inflammatory therapeutics (20, 21). Small molecule Syk inhibitors are currently in clinical development as therapies for autoimmune disorders such as rheumatoid arthritis (22, 23), immune thrombocytopenia, and autoimmune hemolytic anemia. Safety reports from clinical trials demonstrate an increased infectious risk in patients treated with inhibitors of Syk and other tyrosine kinase inhibitors (24). While Syk does play a role in many innate immune cells, its specific role in neutrophil-fungus interactions has not been elucidated. In macrophages, Syk is an important signaling factor in reactive oxygen species (ROS) production, autophagy, and phagosomal maturation, leading to augmented fungicidal activity (25–27). Syk also activates the CARD9 pathway and mediates NLRP3 inflammasome assembly, which can contribute to innate antifungal defense (28–31). Lastly, Syk-dependent signaling can also trigger dendritic cell maturation, CD4^+^ T-cell differentiation, and induction of a cascade of inflammatory cytokines bridging innate and adaptive antifungal activities (31–34). Thus, it is of crucial importance to better understand the possible outcomes of Syk modulation in the context of host immunity against infection.

Neutrophils, or polymorphonuclear cells (PMNs), are innate immune cells that constitute the first line of defense against bacterial and fungal pathogens (17). PMNs employ a variety of effector mechanisms to control *Candida*, including production of ROS, chemokines, cytokines, degradative enzymes, neutrophil extracellular traps (NETs), and phagocytosis (35, 36). Syk has also been implicated in important functional roles in neutrophils against a variety of bacterial and fungal pathogens. Syk-deficient neutrophils were previously shown to fail to undergo respiratory burst, degranulation, or spreading to integrin signaling and to be incapable of generating ROS intermediates in response to FcγR engagement (37, 38). In the absence of Syk, PMNs show impaired exocytosis of secondary and tertiary granules, reduced cytokine release, impaired NET formation, and very poor activation of the NADPH oxidase in response to bacteria, including staphylococci and Escherichia coli (39). In studies of neutrophil fungal interactions with *Aspergillus*, Syk-deficient neutrophils were previously shown to be defective in phagocytosis and killing as well as in ROS production (40, 41). Syk has also been shown to be indispensable for β-glucan-induced NET formation in neutrophils (42). However, the specific role of Syk in neutrophil-*Candida* interactions has not been well characterized.

Despite evidence suggesting a crucial role for Syk in PMNs, elucidation of a precise role for Syk in neutrophils has been challenging for a variety of reasons. Deletion of Syk is embryonic lethal. Early studies on Syk-deficient neutrophils have relied on implantation of chimeric Syk-deleted liver cells into wild-type recipients resulting in *syk* elimination within all hematopoietic cells (37, 40). Other studies use murine models with cell lineage-specific deletion of Syk resulting in *syk* elimination in mature neutrophils or monocytes (39, 42). The short life span and terminally differentiated state of PMNs present major obstacles to defining the molecular mechanisms responsible for their responses to *Candida* (43). To circumvent the limitations posed by primary neutrophils, we used a granulocyte-monocyte progenitor (GMP) cell line that was conditionally immortalized by expression of an estrogen receptor-homeobox B8 (ER-HoxB8) gene fusion as described previously (44, 45). The media for these cells contains stem cell factor (SCF) and estradiol, which permits nuclear translocation of the ER-HoxB8 fusion protein, resulting in conditional maturation arrest at the GMP stage. Removal of estradiol from the cell media allowed synchronous differentiation of the GMP into mature neutrophils (46). This method of gene manipulation also permits a complete deletion of the Syk gene in a neutrophil-specific manner.

In this study, we sought to determine the role of Syk in PMN responses against three major human-pathogenic fungal pathogens, C. albicans, C. glabrata, and C. auris. We show that Syk is essential in neutrophil killing of all three *Candida* species. We further demonstrate that genetic elimination or chemical inhibition of Syk in neutrophils results in diminished tumor necrosis factor alpha (TNF-α) and ROS production. Our results indicate that neutrophils lacking Syk have decreased mechanical responses, such as phagocytosis, NETosis, and swarming, to all three *Candida* species. Together, these data suggest that the loss of Syk results in global defects in neutrophil function against *Candida* species.

## RESULTS

### Primary neutrophils require Syk to eliminate *Candida* species.

**FIG 1:**
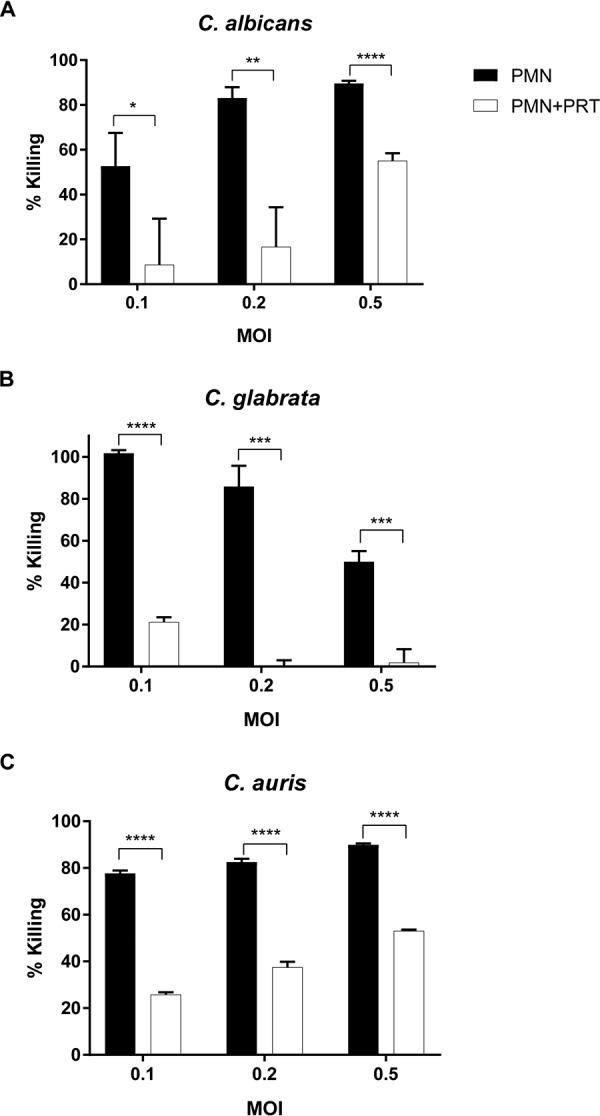
Primary neutrophil killing of *Candida* species is Syk dependent. Primary mouse bone marrow-derived neutrophils were coincubated with C. albicans (2 h) (A), C. glabrata (24 h) (B), and C. auris (6 h) (C) in the absence (PMN) or presence (PMN+PRT) of 2 μM Syk inhibitor PRT. Yeast viability after coincubation was determined by the use of PrestoBlue dye. *, *P* ≤ 0.05; ***, *P* ≤ 0.001; ****, *P* ≤ 0.0001. Data represent results from a minimum of three independent experiments.

Bone marrow-derived primary mouse neutrophils were evaluated for their ability to eliminate C. albicans, C. glabrata, and C. auris *in vitro*. Primary neutrophils were coincubated with yeast at the indicated multiplicities of infection (MOIs) and evaluated for fungicidal activity using PrestoBlue viability indicator dye as previously described (47, 48). Primary neutrophils effectively controlled C. albicans (Fig. 1A) and C. glabrata (Fig. 1B), as well as C. auris (Fig. 1C).

To evaluate the role of Syk, the chemical inhibitor PRT062607 (PRT) (2 μM) was included in the neutrophil and *Candida* coculture. PRT had minimal effect on the viability of yeast or neutrophils alone (viability of >95% by acridine orange [AO] and propidium iodide [PI] staining; data not shown). Interestingly, PRT suppressed the ability of primary neutrophils to kill all three *Candida* species across multiple MOIs (Fig. 1), demonstrating the importance of Syk for neutrophil fungicidal activity.

### Syk-deficient neutrophils lack the ability to kill *Candida*.

**FIG 2:**
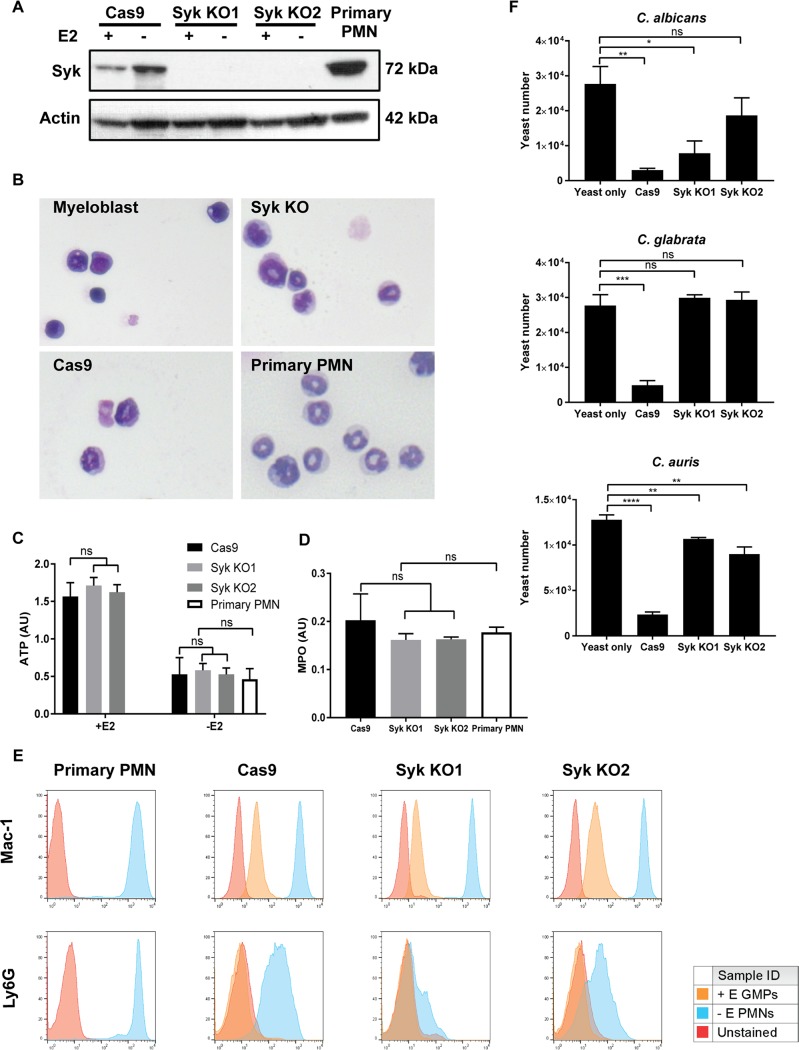
Syk-deficient neutrophils lack the ability to kill *Candida* species. (A) Western blotting of Syk expression comparing parental Cas9 neutrophils, primary bone marrow-derived PMNs, and Syk knockout ER-HoxB8 neutrophils generated using CRISPR-Cas9. (B) Wright-Giemsa staining of GMP myeloblasts, Syk KO cells, Cas9 parent cells, and primary bone marrow-derived neutrophils. (C) Cellular ATP levels of Cas9 parent cells, Syk KO PMNs, and primary bone marrow-derived PMNs, as measured by CellTiter-Glo assays. (D) Myeloperoxidase levels of Cas9 parent cells, Syk KO PMNs, and primary bone marrow-derived PMNs, as measured by TMB. (E) Surface expression levels of Ly6G and Mac-1 on Cas9 parent cells, Syk KO cells, and primary bone marrow-derived neutrophils, as measured by antibody staining and flow cytometry. (F) Cas9 and Syk KO neutrophil cell lines were coincubated with C. albicans, C. glabrata, and C. auris, at an MOI of 0.2, to assess killing levels. Yeast viability after coincubation and PMN lysis was determined by PrestoBlue fluorescence. *, *P* ≤ 0.05; **, *P* ≤ 0.01; ***, *P* ≤ 0.001; ****, *P* ≤ 0.0001; ns, not significant. Data represent results from a minimum of three independent experiments. ID, identifier.

To further investigate the role of Syk in neutrophil killing and verify the observations made with small-molecule inhibitors, genetic Syk knockout (KO) neutrophil cell lines were generated using CRISPR single guide RNA (sgRNA) targeting of Syk in a GMP neutrophil cell line constitutively expressing Cas9. Western blotting demonstrated upregulation of Syk protein expression as cells matured from progenitors (with estradiol [+estradiol]) to terminally differentiated neutrophils (without estradiol [−estradiol]) (Fig. 2A). Syk knockout 1 (KO1) and Syk KO2, two independent cell lines with loss of Syk, were generated; Western blotting confirmed the absence of Syk expression in either the progenitor stage or mature neutrophil stage (Fig. 2A). Despite lacking Syk, the knockout cell lines were capable of differentiation to fully mature neutrophils, demonstrating morphologies by Giemsa staining (Fig. 2B) and ATP production (Fig. 2C) that were similar to those seen with bone marrow-derived primary neutrophils. In addition, Syk knockout cells expressed myeloperoxidase activity levels that were similar to the levels seen with the parental Cas9 neutrophils (Fig. 2D) as well as equivalent levels of surface expression of Ly6G and Mac-1 (Fig. 2E). Similarly to the results seen with chemical inhibition using PRT, Syk-deficient PMN demonstrated significantly attenuated fungicidal activity against C. albicans, C. glabrata, and C. auris (Fig. 2F).

### Syk regulates neutrophil cytokine and ROS production in response to *Candida*.

**FIG 3:**
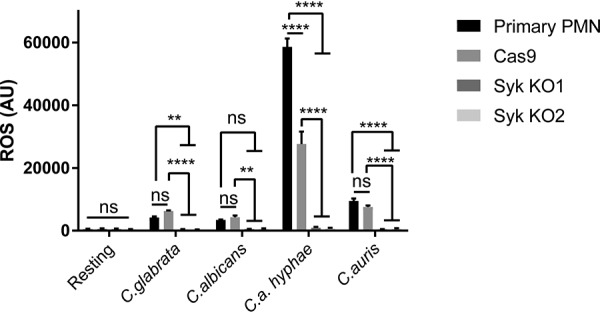
Syk-deficient neutrophils are unable to produce ROS. Primary PMN, Cas9, and two Syk KO neutrophil cell lines were coincubated with heat-killed *Candida* species for 2 h. Values expressed in arbitrary units (AU) represent total lucigenin luminescence levels corresponding to ROS production. *C.a. hyphae* represents pregerminated C. albicans hyphae. “Resting” represents vehicle only. **, *P* ≤ 0.01. ****, *P* ≤ 0.0001. Data represent results from a minimum of three independent experiments.

We next evaluated the role of Syk in neutrophil cytokine and ROS responses to *Candida*. Neutrophils were stimulated with heat-killed *Candida*, and production of ROS was measured by lucigenin luminescence. Following 2 h of stimulation, wild-type neutrophils produced robust ROS levels following stimulation with C. albicans hyphae and, to a lesser extent, with C. auris and C. glabrata (Fig. 3). Syk-deficient neutrophils, on the other hand, did not produce ROS in response to any *Candida* species.

**FIG 4:**
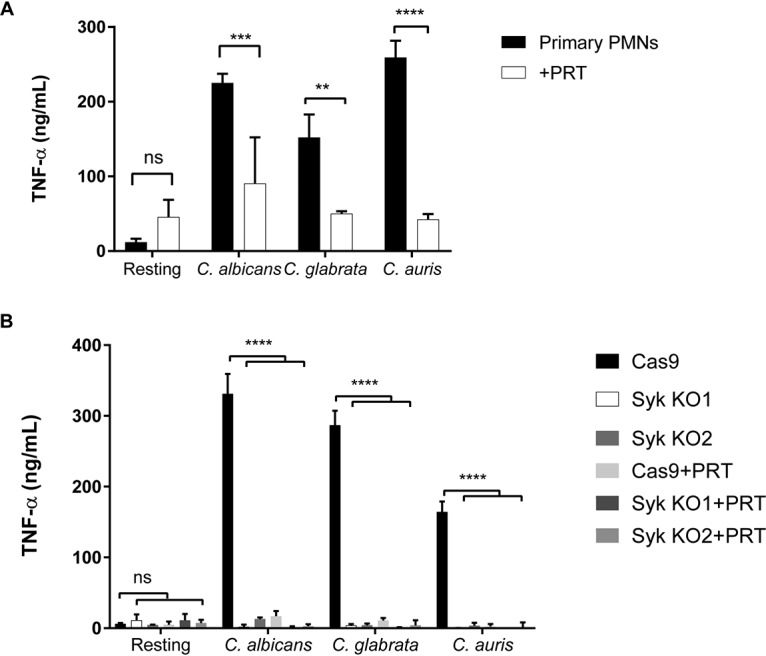
Neutrophil TNF-α production in response to *Candida* species is Syk dependent. Primary mouse bone marrow-derived neutrophils (A) and Cas9 and two Syk knockout neutrophil cell lines (B) were coincubated with heat-killed C. albicans, C. glabrata, and C. auris, at an MOI of 10. Where indicated, 2 μM Syk inhibitor PRT was added. The TNF-α concentration in the supernatant was measured by ELISA. **, *P* ≤ 0.01; ***, *P* ≤ 0.001; ****, *P* ≤ 0.0001; ns, not significant. Data represent results from a minimum of three independent experiments.

To evaluate TNF-α production, neutrophils were stimulated with heat-killed C. albicans, C. glabrata, and C. auris, at a ratio of 10 yeast cells to 1 PMN, and supernatant was analyzed for cytokine stimulation by enzyme-linked immunosorbent assay (ELISA). Primary bone marrow-derived neutrophils produced TNF-α against all three *Candida* species, but production decreased significantly in the presence of 2 μM Syk inhibitor PRT (Fig. 4A). Syk knockout neutrophils also produced much lower levels of TNF-α against *Candida* species than the parental Cas9 neutrophil control (Fig. 4B). The addition of PRT to Syk KO PMN did not have an additional effect on TNF-α production. These data confirm that the production of ROS and secretion of TNF-α in neutrophil responses to *Candida* are dependent on the presence of Syk.

### Syk regulates neutrophil mechanical responses against *Candida*.

**FIG 5:**
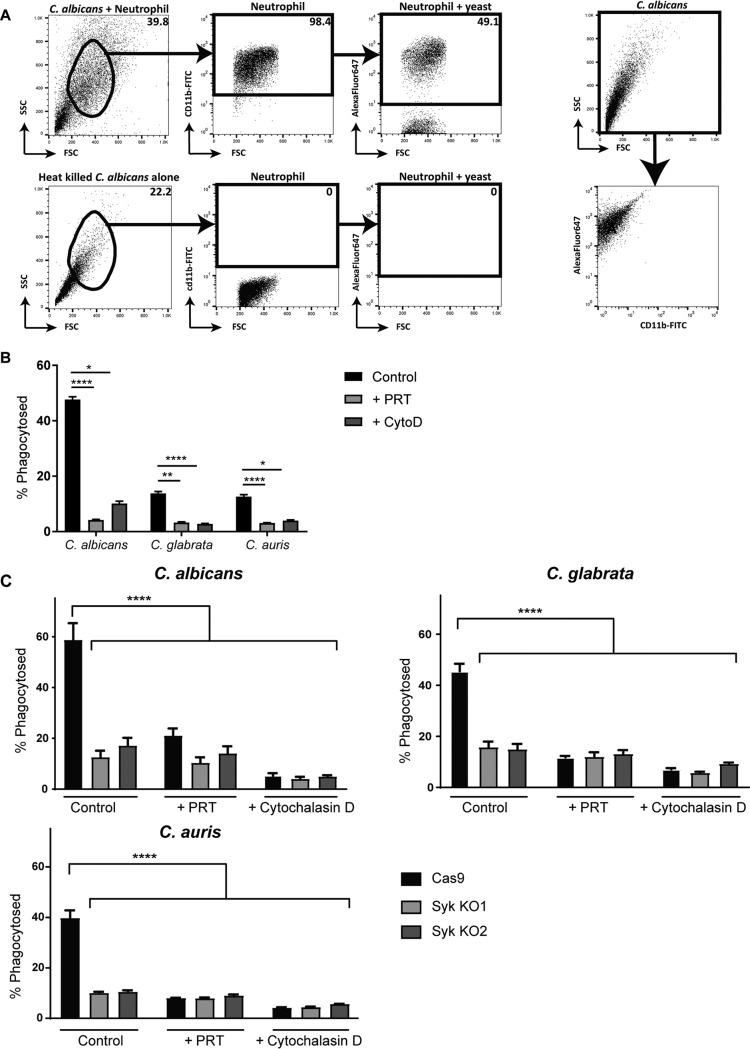
Syk-deficient neutrophils have reduced phagocytosis of *Candida* species. (A) Gating scheme for calculated percentage of neutrophils that have phagocytosed AF647 labeled heat-killed C. albicans by flow cytometry. FSC, forward scatter; SSC, side scatter. (B) Primary mouse bone marrow-derived neutrophils, and (C) Cas9 and two Syk knockout neutrophil cell lines coincubated for phagocytosis with heat-killed and AF647 labeled C. albicans, C. glabrata, and C. auris, at a ratio of 3. Where indicated, 2 μM Syk inhibitor PRT or 30 μM cytochalasin D was added. Phagocytosis events were measured by flow cytometry for AF647 fluorescence in neutrophils after 5 h. *, *P* ≤ 0.05; **, *P* ≤ 0.01; ****, *P* ≤ 0.0001. Data represent results from a minimum of three independent experiments.

We then queried the role of Syk in neutrophil mechanical responses against *Candida*, including phagocytosis, swarming, and NET formation. Neutrophils were coincubated with heat-killed, fluorescently labeled C. albicans, C. glabrata, and C. auris. Phagocytosis was measured by flow cytometry monitoring the uptake of the AF647-labeled yeast with neutrophils (Fig. 5A). Cytochalasin D, an actin polymerization inhibitor, was included as a control for nonphagocytic associations. Primary bone marrow-derived neutrophils phagocytosed all three *Candida* species to different extents by 30 min. In the presence of 2 μM Syk inhibitor PRT, phagocytosis of *Candida* species was almost entirely inhibited to the same activity level as that seen with cytochalasin D (Fig. 5B). Similarly, Syk knockout neutrophils showed decreased phagocytosis of *Candida* species compared to the parental Cas9 neutrophils (Fig. 5C).

**FIG 6:**
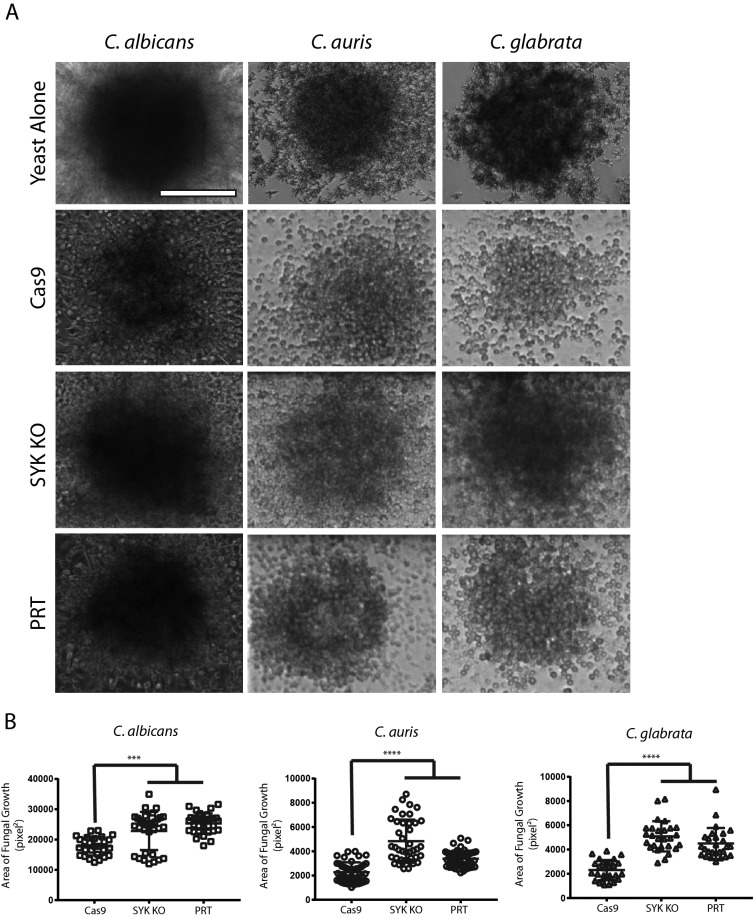
Syk-deficient neutrophils are unable to restrict fungal growth during swarming. (A) C. albicans, C. glabrata, or C. auris cells were patterned on microscale arrays of poly-l-lysine/Zetag and incubated with wild-type Cas9 neutrophils (Cas9), Syk KO neutrophils (SYK KO), Cas9 neutrophils treated with PRT (PRT), or without any cells (Yeast Alone). Bright-field images taken after 9.5 h of incubation are shown for each condition. Fungi grew well on the arrays when incubated alone (first row), but Cas9 cells (second row) were able to restrict fungal growth. Syk KO cells (third row) and PRT-treated cells (fourth row) were less capable of containing fungal growth after swarming. (B) The area covered by fungal growth is quantified as “Area (pixel^2^)” on the *y* axis. Data represent comparisons of Cas9 (wild-type), Syk KO, and PRT-treated Cas9 conditions. *n* = ≥27 swarms across three independent experiments. *, *P* ≤ 0.05; **, *P* ≤ 0.01; ****, *P* ≤ 0.0001 (ANOVA with Tukey’s posttest for C. albicans and C. auris, Kruskal-Wallis for C. glabrata). Scale bars, 100 μm.

To evaluate neutrophil swarming, representing a coordinated multicellular response to a pathogen, C. albicans, C. glabrata, and C. auris were plated in clusters on glass slides and neutrophil behavior was observed using time-lapse light microscopy. While Cas9 cells swarmed around yeast clusters of all three species and effectively controlled yeast proliferation, use of chemical inhibition of Syk with PRT or Syk-deficient cells revealed evenly distributed cells throughout the yeast cluster, representing poor yeast control (Fig. 6A). At 16 h post swarming, yeast clusters were better controlled, with smaller surface areas of fungal growth in the presence of parental, wild-type Cas9 neutrophils, while the Syk-deficient neutrophils resulted in poor control and larger areas of fungal growth (Fig. 6B).

**FIG 7:**
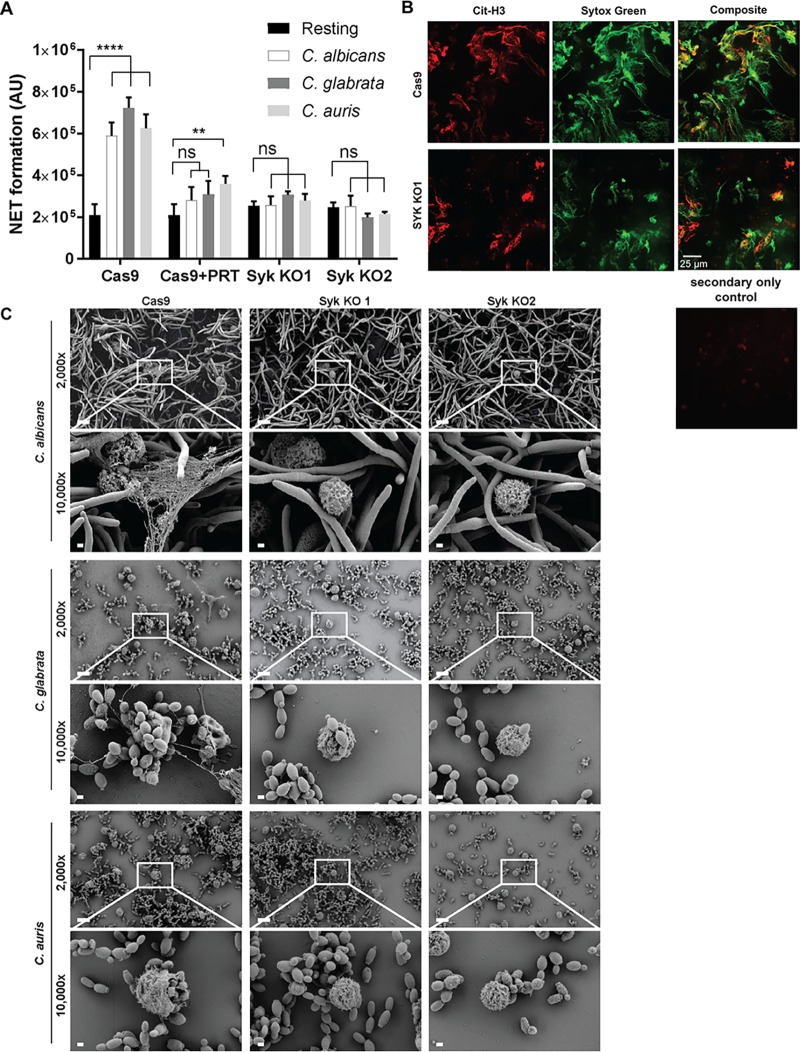
Syk-deficient neutrophils are unable for form NETs. (A) Cas9 and two Syk KO neutrophil cell lines were coincubated with *Candida* species at an MOI of 10. Where indicated, 2 μM Syk inhibitor PRT was added. Arbitrary units (AU) represent Sytox fluorescence corresponding with NET formation. **, *P* ≤ 0.01; ****, *P* ≤ 0.0001; ns, not significant. (B) NETs formed by Cas9 and Syk KO neutrophils were stained with Sytox green and anti-Cit-H3 histone-specific antibody and visualized by fluorescence microscopy. Scale bar, 25 μm. (C) Scanning electron microscopy (SEM) images of neutrophil cell lines coincubated with *Candida* species. Scale bars, 10 μm for the ×2,000 image and 1 μm for the ×10,000 image. Data represent results from a minimum of three independent experiments.

To visualize formation of NETs, neutrophils were coincubated with C. albicans, C. glabrata, and C. auris at an MOI of 10. NET formation was measured by Sytox fluorescence, demonstrating the release of extracellular DNA from wild-type neutrophils following *Candida* stimulation but minimal NET formation in the Syk-deficient cells (Fig. 7A). The citrullinated-histone-specific antibody (Ab) Cit-H3 was used to label NETs in conjunction with Sytox staining and was visualized by fluorescence microscopy (Fig. 7B). Electron microscopy images confirmed NET formation in wild-type Cas9 neutrophils but not Syk-deficient PMN in response to *Candida* (Fig. 7C).

## DISCUSSION

While Syk is an important kinase in innate immune cells, its exact role in neutrophil fungicidal activity has not been completely determined. Here, we demonstrated that multiple critical neutrophil molecular mechanisms responsible for *Candida* control and elimination, including ROS and cytokine production, phagocytosis, NET production, and swarming, are Syk dependent. Chemical inhibition or genetic Syk deficiency results in widespread neutrophil functional defects in response to C. albicans, C. glabrata, and C. auris. Of note, the loss of fungicidal activity was most pronounced in response to C. glabrata, suggesting that Syk may have differential roles depending on the fungal species, most likely related to types of pattern recognition receptors activated by each *Candida* species. Interestingly, our study suggests that mouse neutrophils kill the South American clade of C. auris very effectively, despite other reports demonstrating significant C. auris resistance against human neutrophil killing (49). These observations likely reflect differences between human and mouse neutrophils, as well as differential responses to C. auris clades, although further investigation will be required to elucidate these potential mechanisms.

We explored how loss of Syk results in dysfunctional neutrophil responses to *Candida.* Neutrophils are known to produce abundant levels of ROS, a necessary step for neutrophils to form NETs. In addition, PMNs generate cytokines, including TNF-α, when they encounter *Candida* species also serving as a neutrophil activator (50). Our results demonstrate a reduction of both ROS and TNF-α when Syk is chemically inhibited or genetically deleted from neutrophils. We posit that Syk-dependent receptors are critical for activation of the recognition and response pathways responsible for the core neutrophil functions against *Candida* species.

Swarming is a cooperative and exponential migratory behavior by which neutrophils can protect healthy tissue by rapidly controlling sites of infection (51). Neutrophil swarming occurs in models of sterile tissue damage as well as in response to fungal pathogens, including Cryptococcus neoformans and C. albicans, and is dependent on the presence of leukotriene B4 (LTB4) (51–53). We found that neutrophils swarm around clusters of *Candida* species restricting their growth, and that this ability is compromised in Syk-deficient cells. Thus, in addition to altering lectin receptor signaling, it is likely that Syk plays a role in chemotaxis and locomotion in response to chemoattractants, such as LTB4, a central requirement of swarming (52), though this will require further investigation.

We ensured that the genetic elimination of Syk from the ER-HoxB8 conditionally immortalized neutrophils did not result in a loss of viability that would confound our findings. The Syk-deficient cells demonstrated normal differentiation (as assayed by morphology and cell surface marker expression) as well as preservation of energy production (ATP) and myeloperoxidase (MPO), a central feature of mature neutrophil granules (54). These data suggest that neutrophil dysfunction related to the loss of Syk is not due to maturational cues or differentiation of GMP progenitors into neutrophils.

A limitation of our conditionally immortalized GMP cell line is its restriction to mouse neutrophils. While functions of human and mouse neutrophils are highly conserved, some differences do exist. For example, there are differences in the content of the neutrophil granules, where human PMN have defensins but mouse neutrophils do not (55, 56). They differ in their levels of expression of myeloperoxidase, where murine neutrophils have just 10% to 20% the level of myeloperoxidase activity found in human neutrophils (56). Human neutrophils also have a different propensity for NETosis, where murine neutrophils are less efficient at NET formation and, when made, are more compact than human NETs (57, 58). However, chemical inhibition of Syk in human neutrophils also results in reduced inflammatory cytokine production (59), phagocytosis (60), and ROS production and NET release (61), suggesting that Syk plays similar roles in the two species and that many of our findings in the primary murine neutrophils and cell lines will likely translate to humans.

Our data illustrate an essential role for Syk in neutrophil fungicidal activity against *Candida* species. Primary neutrophils treated with PRT and Syk KO conditionally immortalized PMN lines demonstrated concordant results. Syk appears to influence a variety of pathways, including ROS generation, cytokine production, phagocytosis, swarming, and NET formation. Our studies were limited to *Candida* species necessitating further work to understand the role of Syk when neutrophils engage with other fungi such as dimorphic species and molds causing primary human mycoses. These data raise awareness with respect to future patients who may be treated with Syk inhibition for autoimmune diseases and their increased susceptibility to invasive fungal disease.

## MATERIALS AND METHODS

### Reagents.

PRT062607 (PRT), a specific Syk inhibitor, was purchased from Selleckchem (Houston, TX). Nonidet P-40 (NP-40) was purchased from American Bioanalytical (Natick, MA). Lipopolysaccharide–K-12 (LPS–K-12) was purchased from InvivoGen (San Diego, CA). Cell culture media included liquid YPD (1% yeast extract, 2% peptone, 2% dextrose) (Sigma-Aldrich); liquid RPMI-MOPS (RPMI 1640 containing 2% glucose and 0.165 M MOPS [morpholinepropanesulfonic acid], buffered at pH 7); and complete RPMI (RPMI 1640 with 2 mM l-glutamine, 10% heat-inactivated fetal bovine serum, and 1% penicillin-streptomycin; Thermo Fisher Scientific, Waltham, MA). Stem cell factor (SCF) was derived from conditional media of SCF-overexpressing Chinese hamster ovary (CHO) cells (44, 62). SCF conditional medium was tested for activity, filtered, and stored at –80°C. Inbred C57BL/6 mice (Jackson Laboratory, Bar Harbor, ME) were housed in a specific-pathogen-free facility at Massachusetts General Hospital (MGH). All animal experiments were approved by the MGH Institutional Animal Care and Use Committee. For antibodies, phycoerythrin (PE)-labeled monoclonal antibody (MAb) anti-Mac-1 (CD11b) and allophycocyanin (APC)-labeled monoclonal antibody anti-Ly6G were purchased from BioLegend (San Diego, CA).

### Primary neutrophils.

Primary neutrophils were isolated as previously described (46). Male and female mice used for bone marrow harvests were at least 8 weeks old. Briefly, fresh bone marrow was harvested from mouse femurs and tibias, treated with 0.2% followed by 1.6% hypotonic sodium chloride solution for 20 s each to remove red blood cells, and purified by density gradient centrifugation.

### Neutrophil cell lines.

To circumvent the limitations posed by primary neutrophils, we used a granulocyte-monocyte progenitor (GMP) cell line that was conditionally immortalized by expression of an estrogen receptor-homeobox B8 (ER-HoxB8) gene fusion as described previously (44). The cell media for these cells contain stem cell factor and estradiol, which permits nuclear translocation of the ER-HoxB8 fusion protein, resulting in a conditional maturation arrest at the GMP stage. The removal of estradiol from the cell medium allows synchronous differentiation of the GMP into mature neutrophils (46). Cas9 GMP cell lines were immortalized from the Cas9 transgenic mouse (63), with expression at high (>98%) efficiency (data not shown). All GMP cell lines were cultured in complete RPMI media containing 2% stem cell factor-conditioned media and 0.5 μM β-estradiol and grown in a humidified incubator at 37°C in the presence of 5% CO_2_.

### Yeast.

Wild-type C. albicans (SC5314) was a gift from Eleftherios Mylonakis (Brown Medical School, Providence, RI). Wild-type C. glabrata (ATCC 2001) was purchased from the American Type Culture Collection (ATCC, Manassas, VA). A sequence-confirmed clinical isolate of C. auris belonging to the South American clade was obtained from the MGH microbiology lab (Boston, MA) (64). Yeast cultures were grown overnight in liquid YPD at 30°C with shaking, washed three times in phosphate-buffered saline (PBS) after collection, counted with a Luna automated cell counter (Logos Biosystems, Annandale, VA), and resuspended in PBS at the desired inoculum.

### CRISPR-Cas9 system and sgRNA design.

To construct the lentiviral sgRNA Cas9 vector, single guide RNAs (sgRNAs) were cloned into lentiCRISPR vector in the BsmBI site (46). Lentivirus production and purification were performed as previously described (65). sgRNAs were designed using the CRISPR tool (66) (http://crispr.mit.edu) to minimize potential off-target effects. lentiCRISPR with sgRNAs targeting Syk were cloned using the following sequences: for Syk sgRNA1, ACAGAGACATACCTGCCAGA; for Syk sgRNA2, GTCTTGGGCTGTACTCCCGG; and for Syk sgRNA3, ACACCACTACACCATCGAGA.

Lentiviral infection was carried out in a fibronectin (Sigma)-coated 12-well plate. GMP cells (2.5 × 10^4^) were infected with Syk sgRNA containing lentivirus by spinoculation (1,000 × *g*, 90 min, 22°C) in the presence of 24 μg/ml Polybrene (Millipore, Burlington, MA).

### PrestoBlue killing assay.

Neutrophils were plated at 1 × 10^5^ cells per well in complete RPMI medium in a 96-well tissue culture plate. PRT (2 μM) was added to pretreat appropriate wells for 20 min at 37°C with 5% CO_2_. After Syk inhibitor pretreatment, various multiplicities of infection (MOIs) of *Candida* species were coincubated with the neutrophils (C. albicans for 2 h, C. auris for 4 h, and C. glabrata for 24 h). A standard curve was generated with serial dilution of yeast cells. Following incubation, all PMNs were lysed with a 1% NP-40 solution containing 10 mM Tris HCl, 150 mM sodium chloride, and 5 mM magnesium chloride, titrated to pH 7.5. After neutrophil lysis, optimized yeast growth medium was added to supplement *Candida* growth (complete RPMI medium for C. albicans, YPD medium for C. glabrata, and RPMI-MOPS for C. auris). Finally, 10% PrestoBlue cell viability reagent (Thermo Fisher Scientific) was added to each well and fluorescence was read every hour for 18 h at 30°C using a SpectraMax i3x reader (Molecular Devices, Sunnyvale, CA) (67). Standard yeast growth curves were generated to accurately determine the number of viable yeasts. Fluorescence was plotted versus time, and the time to the midcurve (inflection point) was determined using GraphPad Prism 7 software (La Jolla, CA). Percent killing was determined as follows: 1 − (viability of yeast plus neutrophils/viability of yeast only).

### Reactive oxygen species (ROS) production.

Reactive oxygen species production was measured as previously described (25). Briefly, neutrophils were plated at 5 × 10^5^ cells per well in complete RPMI medium in 96-well white-walled plates (Grenier Bio-One, Monroe, NC). Cells were placed on ice, and heat-killed *Candida* species were added at an MOI of 10. A lucigenin solution was added to each well for a final concentration of 15 μM lucigenin. Luminescence was measured at 37°C every 5 min for 4 h in a SpectraMax i3x reader (Molecular Devices, San Jose, CA), and the results were expressed as arbitrary luminescence units.

### TNF-α enzyme-linked immunosorbent assay (ELISA).

Neutrophils were plated in 96-well tissue culture dishes at 1 × 10^5^ cells per well in complete RPMI medium and were cocultured with heat-killed *Candida* at an MOI of 10. Where appropriate, wells were treated with Syk inhibitor PRT at a concentration of 2 μM. After 24 h of stimulation at 37°C with 5% CO_2_ supplementation, the concentration of TNF-α in the supernatant was measured with ELISA per the instructions of the manufacturers (Duoset; R&D Systems, Minneapolis, MN, and BD Pharmingen, San Jose, CA).

### Phagocytosis.

To assess phagocytosis, neutrophils were coincubated with AF647-stained heat-killed *Candida* yeast cells at a ratio of 3 yeast cells per PMN for 5 h at 37°C in a 1.5-ml tube. Samples were fixed in 10% formalin. Neutrophils were labeled with CD11b-fluorescein isothiocyanate (CD11b-FITC), and samples were divided into three wells of a 96-well U-bottom plate. Where appropriate, cells were pretreated with the Syk inhibitor PRT at a concentration of 2 μM or with cytoskeletal inhibitor cytochalasin D (Sigma) at a concentration of 30 μM. Flow acquisition was done with a high-throughput plate adaptor attached to a FACSCalibur flow cytometer (Becton, Dickinson, San Jose, CA, USA), using CellQuest software (Becton, Dickinson). Percent phagocytosis was measured by selecting for doubly positive CD11b^+^ and AF647^+^ neutrophils. Flow data were analyzed using FlowJo 10 software (FlowJo, Ashland, OR).

### Western blotting.

Neutrophils were lysed in Pierce radioimmunoprecipitation assay (RIPA) buffer (Thermo Fisher Scientific) containing protease inhibitors (cOmplete Mini; Roche Diagnostics, Indianapolis, IN) and sodium orthovanadate (New England BioLabs, Ipswich, MA). Proteins were resolved by SDS-PAGE under reduced conditions and transferred onto a polyvinylidene difluoride (PVDF) membrane. Membranes were blocked in Tris-buffered saline–1% Tween (TBS-T)–5% nonfat milk. Total Syk protein was detected with rabbit MAb D3Z1E anti-Syk (Cell Signaling, Danvers, MA) (1:1,000). The blots were subsequently reacted with mouse MAb AC-15 anti-β-actin (Sigma, St, Louis, MO) (1:200,000) to verify equivalent loading levels.

### Cellular ATP level.

Live cells (2 × 10^4^ per well) were plated in PBS on a 96-well white-walled plate. CellTiter-Glo (Promega, Madison, WI) was added at a 1:1 volume ratio, and the cells were incubated for 5 min in the dark and assessed for total luminescence using a SpectraMax i3x reader.

### Myeloperoxidase (MPO) activity.

Purified live neutrophils (5 × 10^5^ per well) were plated in PBS on a 96-well tissue culture plate and serially diluted. Triton X-100 (Sigma, St. Louis, MO) was added at 0.1% for cell lysis. A TMB (3,3′,5,5′-tetramethylbenzidine) substrate reagent set (BD Biosciences, San Jose, CA) was then added at a 1:1 volume ratio to the lysed cells. Endpoint absorbance was read at 605 nm on the SpectraMax i3x reader, and the results were expressed as arbitrary units corresponding to MPO activity levels.

### Flow cytometric cell surface marker analysis.

Cells (1 × 10^5^) were labeled with PE-labeled or APC-labeled MAb for 30 min at 4°C in phosphate-buffered saline (PBS)–1% fetal bovine serum (FBS)–1 mM EDTA, washed, and resuspended in the same buffer. Flow cytometry data were acquired with the CellQuest program on a FACSCalibur. Live cells were gated for analysis of forward and side scatter signals. Surface marker expression was measured as geometric mean fluorescence and analyzed using FlowJo 10 software.

### Wright-Giemsa staining.

Wright-Giemsa staining was performed as previously described (44). Cytocentrifuge preparations of cells (Thermo Shandon, Pittsburgh, PA) were stained 4 min in Wright stain, followed by 12 min in 20% Giemsa stain. Staining was visualized with light microscopy.

### Neutrophil swarming microscopy.

Utilizing a microarray printing platform (Picospotter; PolyPico, Galway, Ireland) and arrays of spots prepared with a solution of poly-l-lysine (Sigma-Aldrich, Saint Louis, MO), ZETAG and FITC (added to visualize the spots by microscopy) were printed onto ultraclean glass slides (Fisher Scientific, Hampton, NH). Slides were screened for accuracy and then dried for at least 2 h before use. ProPlate 16-well plates (Grace Bio-labs, Bend, OR) were attached to the glass slides with printed arrays. A 50-μl volume of the desired target, consisting of live C. albicans, C. glabrata, or C. auris inocula suspended in PBS, was added to each well, and each suspension was incubated with rocking for 5 to 10 min. Following incubation, the wells were thoroughly washed out with water to remove unbound yeast from the glass surface. Wells were screened to ensure appropriate patterning of targets onto the spots with minimal nonspecific binding before use. Swarming was observed using a Nikon Ti-E microscope equipped with an electron-multiplying charge-coupled-device (EM-CCD) camera (Hamamatsu; C9100-13). The microscopy chamber, housed in a Plexiglas environmental chamber, was humidified and maintained at 37°C (68). The swarming targets to be observed during the experiment were selected and saved using the multipoint function in Metamorph (Molecular Devices, Downingtown, PA) prior to loading. Neutrophils (5 × 10^5^) were then added to each well. All selected points were optimized for perfect focus before launching of the experiment. For the GMP cells treated with 2 μM PRT, the neutrophils were preincubated for 30 min before use. Swarming area analysis was done by manually outlining the swarms or areas of fungal growth in ImageJ software. Images were analyzed using Adobe Creative Cloud Photoshop and Illustrator (Adobe, San Jose, CA).

### Neutrophil extracellular trap (NET) formation quantification.

Neutrophils were plated at 5 × 10^5^ cells per well in complete RPMI medum in a 96-well tissue culture plate. Where appropriate, wells were treated with PRT at a concentration of 2 μM. *Candida* species at an MOI of 10 were coincubated with the neutrophils for 1 h at 37°C. After the incubation, Sytox red (Invitrogen, Carlsbad, CA) was added at a final concentration of 160 nM to detect extracellular DNA. Sytox red fluorescence was read every hour for 18 h at 30°C using a SpectraMax i3x reader, and the results were expressed as arbitrary fluorescence units. Staining for NET histones was carried out as previously described (69). Briefly, neutrophils were incubated with C. albicans hyphae or C. glabrata and C. auris yeast cells for 3 h. Samples were then stained with anti-histone H3 citrulline R2+R8+R17 (Abcam, Cambridge, MA; 0.014 mg/ml) followed by donkey anti-rabbit IgG Cy3 (Jackson Immunoresearch, West Grove, PA; 0.0075 mg/ml) secondary antibody. Samples were then imaged at ×40 on Nikon Ti-E microscope.

### Scanning electron microscopy.

A coverslip model was used for scanning electron microscopy experiments, as previously described (70, 71)*. Candida* yeasts (1 × 10^6^) were added to poly-l-lysine-treated plastic 13-mm-diameter coverslips (Thermanox; Thermo Fisher Scientific, Waltham, MA) and incubated for 1 h at 30°C. Neutrophils (5 × 10^5^) were added to wells, and cocultures were incubated for 4 h at 37°C. After washing was performed, coverslips were fixed overnight in 4% formaldehyde–1% glutaraldehyde, followed by washing and treatment with 1% osmium tetroxide. Samples were then washed, dehydrated through a series of ethanol washes followed by critical-point drying, and coated with a 14-nm-thick platinum layer. Microscopy was completed on a LEO 1530 scanning electron microscope at 3 kV.

### Statistics.

Statistical calculations were performed using GraphPad Prism 7 software (La Jolla, CA). Data were analyzed by two-tailed unpaired *t* test or by one-way analysis of variance (ANOVA), where appropriate, and were considered significantly different for *P* values of ≤0.05.
